# The Impact of Clinical and Demographic Factors on High-Risk Patient Classification Frequencies by the EndoPredict Test: A Review and Single-Site Study

**DOI:** 10.3390/cancers18060951

**Published:** 2026-03-14

**Authors:** Gabriele Raciti, Paolo Fontana, Stefano Forte

**Affiliations:** Mediterranean Institute of Oncology, 95029 Viagrande, Italy; gabriele.raciti@grupposamed.com (G.R.); paolo.fontana@grupposamed.com (P.F.)

**Keywords:** breast cancer, EndoPredict, multigene expression panels, population intrinsic risk, breast cancer prognosis, adjuvant chemotherapy

## Abstract

**Background/Objectives**: EndoPredict is a second-generation prognostic assay for estrogen-receptor-positive, HER2-negative breast cancer that integrates molecular and clinical parameters for risk stratification. Multiple studies have reported its clinical utility, while differences in the proportion of patients classified as high- or low-risk have been observed across cohorts. This study aimed to characterize clinical, pathological, and demographic factors associated with these differences. **Methods**: We conducted a descriptive review of 17 published studies and analyzed a single-institution cohort of 140 patients. Associations between clinicopathological variables and high-risk classification were assessed, including tumor size, lymph node status, histological grade, Ki-67 expression, and reproductive and demographic factors. Differences in inclusion criteria and cohort characteristics were also examined. **Results**: Tumor size and lymph node involvement emerged as primary determinants of high-risk classification. A high histological grade and Ki-67 levels above 25% were significantly associated with high-risk status (*p* < 0.001). Conversely, age, age at menarche, menopausal status, Body Mass Index, progesterone receptor expression, molecular subtype, and histological type showed no significant association. A higher number of pregnancies correlated with a lower frequency of high-risk classification (*p* < 0.01). Heterogeneity in risk distribution across studies was largely attributable to differences in tumor size, nodal involvement, and histological grade. Additional variability was associated with inclusion criteria, sample selection, and regional demographic characteristics. **Conclusions**: Variability in EndoPredict risk classification reflects both tumor biological features and population-specific factors. These findings emphasize the importance of interpreting genomic risk scores within their clinical and demographic context and support the comparison of risk distributions across heterogeneous patient cohorts.

## Simple Summary

Gene expression tests such as EndoPredict are widely used to support treatment decisions in hormone-receptor-positive breast cancer. However, different studies often report varying proportions of patients classified as high- or low-risk, which may raise concerns when local results differ from published data. In this work, we combined a descriptive review of published studies with data from our own patient cohort to better understand the reasons behind these differences. We found that tumor size, lymph node involvement, histological grade, and tumor proliferation strongly influence risk classification, while several demographic and reproductive factors play a more limited role. Our results indicate that variability in risk distributions mainly reflects differences in patient populations and case mix across cohorts. These findings help contextualize the divergent risk frequencies observed in clinical practice and support confidence in the robustness of EndoPredict across diverse clinical settings.

## 1. Introduction

The latest available estimates from the World Health Organization’s cancer agency and the International Agency for Research on Cancer report 2.3 million new cases of breast cancer (BC) and nearly 700,000 deaths in 2022 [1]. BC is the second most common cancer by incidence, following lung cancer, and the fourth leading cause of cancer-related mortality after lung, colorectal and liver cancers [2]. While BC incidence has stabilized in recent years due to better population screening programs, earlier-stage diagnosis, and therapeutic strategies, its mortality rate has declined, at least in the high-income countries [3].

BC is not only histologically but also molecularly heterogeneous and can be classified into different subtypes based on the expression of immunohistochemical markers or gene expression profiles obtained through different techniques. Intrinsic subtypes, first defined by Sørlie et al. [4] in 2001 through gene expression analysis, have gained widespread acceptance as a method for classifying breast tumors. Four main categories, in fact, can be discriminated inside invasive BCs: Luminal A and Luminal B types (both hormone receptor(HR)-positive and representing 30–40% and 20–30%, respectively), HER2-positive and basal-like. Each of them displays different immunophenotypic features, depending on the expression of both hormone receptors and human epidermal growth factor receptor 2 (HER2) as well as Ki-67 levels [5,6].

HR+/HER2-BCs, which are the majority of cases diagnosed, generally present a good prognosis after surgery and are typically treated with hormone therapy alone or in combination with chemotherapy, with good response rates in terms of recurrence and survival [7,8]. In order to avoid short- and long-term negative consequences due to overtreatment [9], it is therefore mandatory to better tailor the management of post-surgery BC patients [10]. Such efforts are particularly worthily for those patients who belong to the “intermediate risk” category, which makes indications for chemotherapy treatment challenging. In this scenario, the recent appearance of prognostic genomic testing is advised and supports the therapeutic decision-making process [11].

To date, five multigene tests, despite being fairly different from each other, namely Prosigna^®^, MammaPrint^®^, Oncotype DX^®^, Breast Cancer Index^®^ and EndoPredict^®^, have the greatest clinical validation [12,13,14,15,16]. Each of them, starting from tumor tissue specimens, focuses on the expression of different subset of genes by employing different methodologies and produces recurrence risk scores.

Among them, EndoPredict^®^, whose methodology and clinical validation were first reported by Filipits and colleagues in their work [16], is a second-generation RNA-based test intended for primary female invasive, ER+, HER2-BCs for whom the administration of chemotherapy, in addition to the endocrine one, is questionable. The EndoPredict assay provides prognostic information (both 0–10-year and 5–15-year risks of distant recurrence) as well as the estimated absolute benefit of chemotherapy at 10 years [16,17,18]. It uses real-time PCR to analyze the expression of twelve genes in tumor cells: eight target genes representing ER signaling/cell differentiation and proliferative/cell cycle pathways, three reference genes, and one gene as a DNA contamination control. Based on their expression levels, the test generates a molecular score (EPscore) whose combination with tumor size and axillary lymph node status produces the EPclin score. Because the EPclin score integrates both molecular and clinical variables, differences in the distribution of clinicopathological characteristics across patient cohorts may substantially influence the proportion of patients classified as low- or high-risk. The EPclin cut-off value of 3.32867 distinguishes patients with a low likelihood of recurrence (<3.32867) from those with a high likelihood (≥3.32867), eliminating an “intermediate-risk” category that could otherwise complicate clinical decision-making.

The aim of this review is to evaluate the influence of several intrinsic clinical factors in the observed frequencies of low- and high-recurrence-risk patients by analyzing several BC cohorts subjected to the EndoPredict assay. To achieve this, we analyzed not only tumor size and lymph node status, which are already included in the EPclin score algorithm, but also several other clinical factors. These include age, ER/PR expression status, tumor grade, menopausal status, histological and molecular subtypes, age at menarche, BMI, and number of pregnancies. To better contextualize the variability observed across published data, we categorized the included studies according to whether the majority of patients were classified as low- or high-risk. This descriptive framework was intended to highlight systematic differences in underlying patient and tumor characteristics, which likely account for the heterogeneous frequencies of the EPclin risk groups reported in the literature, rather than to generate new prognostic information. This analysis was conducted across both categories as well as within an independent cohort of patients from our institution. By incorporating these additional factors, our study provides a broader perspective on how differences in patient and tumor characteristics may influence the observed distribution of EPclin risk categories across clinical cohorts.

## 2. Materials and Methods

A literature search was conducted from June 2024 to December 2024 in the PubMed database (https://pubmed.ncbi.nlm.nih.gov/) using the keywords “EndoPredict”, “EndoPredict breast” and “EndoPredict breast cancer” to identify research papers discussing the application of this genomic assay inside different clinical cohorts of breast cancer patients. In addition, cross-referencing of retrieved articles was performed to identify potentially relevant studies and conference proceedings not captured by the initial search. Although not identified, non-English studies were not excluded upstream but considered includable if sufficient data could be extracted.

Titles and abstracts were independently screened by two investigators to identify potentially eligible studies. The full texts of the selected articles were then assessed independently for eligibility. Disagreements were resolved through discussion until consensus was reached; when needed, a third investigator arbitrated.

Studies were considered eligible if they reported the use of the EndoPredict or EPclin score in breast cancer patients and provided information on the proportion of subjects classified as low-risk (LR) and high-risk (HR). Studies were excluded if they analyzed populations outside the intended indication of the EndoPredict assay (e.g., estrogen-receptor-negative tumors, HER2-positive disease, male breast cancer, or stage IV breast cancer), analyzed overlapping cohorts already represented in other publications, or did not report any clinicopathological characteristics of the enrolled patients.

Forty articles spanning from 2011 to 2024 were initially identified as eligible for the aim of the review and were analyzed alongside our cohort (the “IOM cohort”). Specifically, the patients’ enrollment in our center from 2022 to 2024 was consecutively and retrospectively performed. Informed consent was obtained from all the subjects involved in this study, and both demographic and clinicopathological characteristics were collected, categorized and organized in an internal dataset.

The inclusion criteria for patients’ enrollment were as follow: age ≥ 18 years; a diagnosis of pT1-T3 breast cancer with or without lymph node involvement; estrogen-receptor-positive breast cancers; HER2-negative disease; female subjects; and absence of previous chemotherapy (treatment-naïve). Also, almost all enrolled patients are white women, and most of them are European. On the contrary, the following exclusion criteria were included: a diagnosis of stage IV breast cancer; estrogen-receptor-negative breast cancers; HER2-positive disease; presence of a previous chemotherapy regimen; male subjects; and patients affected by tumor recurrence.

The objective of this review was to investigate clinical, pathological, and demographic factors associated with the asymmetric distributions of EndoPredict risk classification across patient cohorts. Therefore, the analysis focused on studies showing a clear predominance of either LR or HR classifications.

From the original forty articles, seven studies were excluded because the difference between the percentages of subjects classified by EPclin score as LR and HR was less than 5%, indicating a near-balanced distribution that could not be assigned to either contrast group. This threshold was used as an operational criterion to define near-balanced cohorts for the contrast analysis. Specifically, in Bertucci et al.’s study [19], 283 patients were LR (51%), while 270 (49%) were HR, and stage IV breast cancers were included as well. In Fitzal et al.’s study [20], among the 1324 enrolled individuals, 641 (48%) were classified as LR and 683 (52%) as HR, while in Sandoval et al.’s study [21], 26 patients were LR (52%) and 24 were HR (48%). The four remaining studies [22,23,24,25] revealed a perfect balance between LR and HR subjects (50% for both categories).

**Figure 1:**
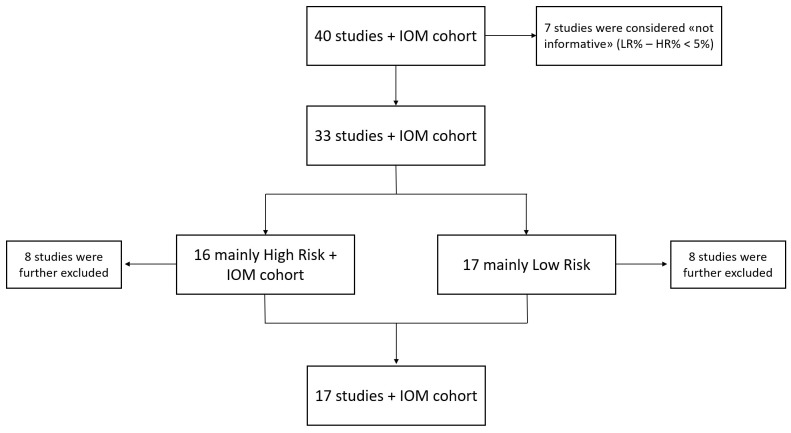
Review flow chart. The above scheme represents the design of this review from the top to the bottom. Abbreviations: IOM: Istituto Oncologico del Mediterraneo; LR: low-risk; HR: high-risk.

The remaining thirty-three studies and the IOM cohort were dichotomized according to the predominant risk classification. Specifically, sixteen articles and our cohort displayed a majority of HR-score subjects (hereafter “HR-group”), while seventeen studies revealed more LR cases than HR ones (referred to as “LR-group”). However, eight studies from both categories were excluded as they exhibited characteristics that could introduce bias in the comparison. These included: studies analyzing the same cohort as other already selected articles [17,18,26,27,28], complete absence of clinical characteristics for enrolled patients [29,30], inclusion of ER-negative [31,32,33,34,35] or presence of HER2-positive breast cancers [36], enrolment of male subjects [29,37,38], or presence of some subjects with unclassified EP and EPclin scores [39]. Excluded studies and the primary reason/s for exclusion are reported in Supplementary Table S1. Consequently, seventeen studies, whose key inclusion criteria are listed in Supplementary Table S2, plus our cohort underwent review analysis [16,40,41,42,43,44,45,46,47,48,49,50,51,52,53,54,55]. Figure 1 illustrates the flow chart used for the review.

To evaluate the robustness of the cohort classification strategy and the choice of the near-balanced threshold, a descriptive sensitivity analysis was conducted by comparing summary measures of selected clinicopathological variables across LR- and HR-predominant cohorts using alternative thresholds to define near-balanced distributions (Δ < 5% and Δ < 10%). Near-balanced cohorts were summarized separately and not included in the primary contrast analysis. The results of this analysis are reported in Supplementary Table S3.

The analyses were performed using GraphPad Prism (v.8.0.1). One-way ANOVA analysis was carried out for tumor size, grade, nodal status and progesterone receptor (PR) expression between the two risk groups. Tukey’s correction was applied to the post hoc analysis. The Mann–Whitney test was applied to compare age of menarche and Body Mass Index, while an unpaired *t*-test was performed to compare the number of pregnancies and Ki-67 expression in IOM patients between LR and HR categories. Conversely, the frequencies of high- and low-risk individuals according to menopausal status, nodal status, tumor grade, PR expression, surrogate intrinsic subtype, and histology subtype were analyzed using Fisher’s Exact test, while the Chi-Square test was applied for tumor size, and estrogen receptor (ER) expression. The normal distribution of the data was evaluated with the Shapiro–Wilk normality test, and the alpha value was set to 0.05 for all tests.

Additionally, to investigate the independent contribution of clinical and biological variables to the EPclin risk classification frequencies, we performed multivariate logistic regression analyses with the binary outcome high-risk vs. low-risk. The variables included were age, number of pregnancies, tumor size (pT category), nodal status, histological grade, and Ki-67. Tumor size (pT1ab), negative nodal status, and grade G2 were used as reference categories. Ki-67 was modeled as both a continuous variable (per 1% increase) and, in an alternative model, as a dichotomous variable using the 20% cut-off, in line with the St. Gallen International Consensus recommendations for the distinction between Luminal A and Luminal B breast cancers.

Multivariate logistic regression models were fitted using the glm() function of the base stats package in R. Odds ratios (ORs) with 95% confidence intervals (95% CIs) were obtained using the broom package (version 1.0.12), and variance inflation factors (VIFs) were assessed with the car package (version 3.1.5) to exclude relevant collinearity. Model performance was evaluated by receiver operating characteristic (ROC) curve analysis and area under the curve (AUC) calculation with the pROC package (version 1.19.0.1). Given the exploratory nature of the study and the limited sample size of the institutional cohort, the number of predictors included in the multivariate model was restricted to key clinicopathological variables known to influence EndoPredict risk classification. No internal validation procedures (e.g., bootstrap or cross-validation) were performed; therefore, the results of the multivariate model should be interpreted as exploratory.

Analyses were performed using complete-case data. Observations with missing values for the variables involved in a given analysis were excluded from that specific analysis. The proportion of missing data was generally low (e.g., 4.3% for intrinsic subtype classification), and therefore no imputation procedures were applied. Again, a *p*-value < 0.05 was considered statistically significant. All analyses were performed in R version 4.5.1 (R Foundation for Statistical Computing, Vienna, Austria).

## 3. Results

As a result of the literature search and the subsequent selection (reported in Section 2), seventeen studies along with the IOM cohort were ultimately analyzed. Notably, the studies include nine articles reporting a majority of subjects with a tested low EPclin score (LR group), and eight studies indicating more HR-classified patients (HR group). Several clinical characteristics have been compared between both groups to understand whether other aspects, beyond tumor size and lymph node status, could influence risk class stratification. Our hospital cohort, referred as the “IOM cohort”, which is made of a majority of high-risk subjects, encompasses 140 patients, and their clinical characteristics are summarized in Table 1.

### 3.1. Age, Age of Menarche, Menopausal Status, Number of Pregnancies, and BMI

**Figure 2:**
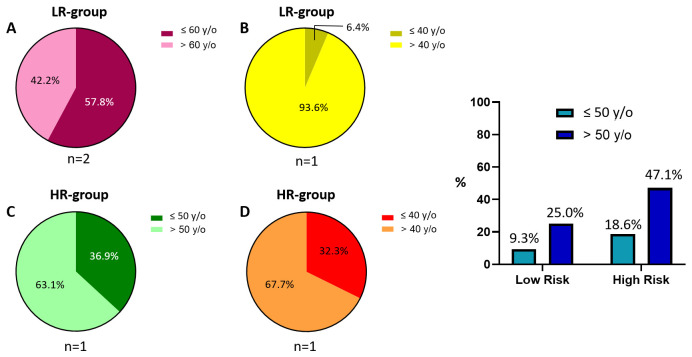
Age. Pie charts of BC patients from the LR studies using 60 y/o (**A**) and 40 y/o (**B**) as the thresholds. Pie charts of BC patients from the HR studies considering 50 y/o (**C**) and 40 y/o (**D**) as the cut-off values. Bar chart of the IOM cohort (**E**). Abbreviations: IOM: Istituto Oncologico del Mediterraneo; BC: breast cancer; LR: low-risk; HR: high-risk; y/o: years old. The “%” symbol on the y-axis refers to the whole IOM cohort. Calculations are unweighted.

Both the LR and HR groups display discrepancies in the chosen cut-off values related to the breast cancer (BC) patients’ ages. Moreover, only a subset of them reports such data: regarding the LR group, two articles [16,49] define “60 years old (y/o)” as a discriminative value (Figure 2A), while “40 y/o” is indicated in the other study [47] (Figure 2B). In contrast, both HR studies [44,55] differ in the used threshold, with “50 y/o” and “40 y/o” as the selected ones (Figure 2C,D). Hence, the indication of a higher presence of older subjects in the HR group compared to the LR one cannot be fully supported due to both the limited number of available studies and the defined ranges.

This trend interestingly aligns with that of our cohort, which is skewed towards individuals over 50 years old (72.1%, Figure 2E). However, when considering the two age groups (≤50 y/o and >50 y/o) separately, our cohort shows an almost double frequency of HR cases compared to LR ones in both groups. Therefore, age is not a significant discriminating factor for patient risk frequency.

**Figure 3:**
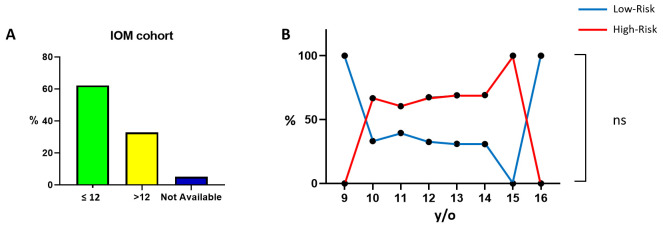
Age of menarche. Bar chart illustrating the IOM cohort distribution with a cut-off value of 12 (**A**). Graphical representation presenting the numbers and relative percentages of LR and HR patients across all considered categories (**B**). Abbreviation: y/o: years old. The “ns” symbol indicates *p* > 0.05; the “%” symbol on the y-axis refers to the whole IOM cohort for subfigure (**A**), while it refers to a subgroup of it for subfigure (**B**) (information that was not available was excluded). Calculations are unweighted.

Next, the following analyzed parameter was the age of menarche. Such data are not mentioned in all seventeen selected articles; therefore, the analysis has been limited to the IOM cohort. By stratifying our patients, we observed that menarche predominantly occurs at the age of twelve, which was selected as the cut-off value (Figure 3A and Table 2). Although the majority of patients in the IOM cohort have an age of menarche at or below twelve, the risk class sub-analysis within the two categories (≤12 and >12) does not reveal a clear trend (Figure 3B). Additionally, while there may be a trend starting from the age of 10, as indicated in Figure 3B, the statistical analysis does not demonstrate a significant association with the age of menarche (*p* = 0.70), even when excluding the 16 y/o value.

The patients’ menopausal condition is partially indicated in both the LR and HR groups, limiting the ability to compare their data with those of our cohort. Only three articles in the LR group [16,41,42] documented this status: in two of them, the entire population considered is composed of post-menopausal subjects, while they are pre-menopausal in the other one (Figure S1A). Conversely, two studies in the HR group [43,55] provide the menopausal status: patients are entirely pre-menopausal in the first study and mixed in the second one (35% and 65%, respectively, Figure S1B). Therefore, due to the low quality and quantity of data provided, no indication can be drawn. The IOM cohort, which predominantly consists of post-menopausal BC subjects (62.1%, Figure S1C), shows the irrelevance of this factor. In fact, nearly identical percentages were obtained from the analysis (*p* = 0.65) across the two inquired categories (Table S5).

**Figure 4:**
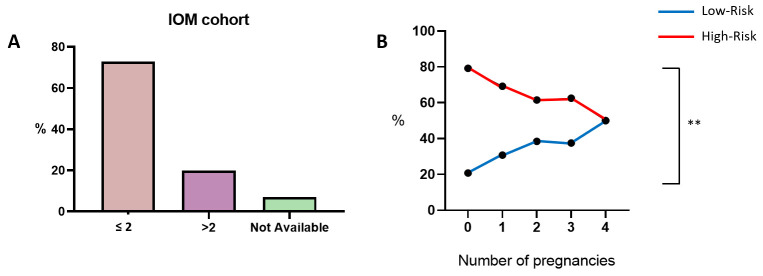
Number of pregnancies. Bar chart of IOM-cohort stratification based on “two pregnancies” as the cut-off value (**A**). Graphical representation displaying the numbers and relative percentages of LR/HR patients among the different recorded numbers of pregnancies (**B**). Abbreviation: IOM: Istituto Oncologico del Mediterraneo. The “**” symbol indicates *p* ≤ 0.01; the “%” symbol on the y-axis refers to the whole IOM cohort for subfigure (**A**), while it refers to a subgroup of it for subfigure (**B**) (information that was not available was excluded). Calculations are unweighted.

In addition to the age of menarche, the number of pregnancies is another underestimated parameter, and no indications have been provided for either the LR or HR group. Following the previous approach, IOM-cohort subjects were stratified based on their number of pregnancies: most patients have had a maximum of two pregnancies (72.9%, Figure 4A); thus, it was chosen as the cut-off value. Even in this case, the sub-analysis of LR/HR case percentages within the two defined categories (≤2 and >2) does not yield any evident indication. However, as the number of pregnancies increases, the relative percentages of HR patient progressively decrease, while there is a corresponding increase in the percentages of LR subjects (Figure 4B and Table 3). Since this analysis reveals a statistically significant tendency (*p* = 0.0027) between number of pregnancies and EPclin results, it suggests that higher parity in our cohort seems to be associated with a lower frequency of EPclin high-risk classification.

Body Mass Index (BMI) is a simple weight-to-height index. It was analyzed in the IOM cohort, which investigated differences within the LR and HR groups. Following the World Health Organization’s guidelines [56], individuals were stratified into four categories, namely underweight, normal, overweight and obese, along with one group that had no available data (Figure S2A). The analysis of these four categories does not reveal any trend, and subsequent statistical evaluation confirms a lack of meaningful insights (Figure S2B, Table S6).

### 3.2. Tumor Stage, Nodal Status, and Grade

Then, tumor stage and nodal status, both pivotal and contributing parameters in defining the EndoPredict Clinical (EPclin) score, along with tumor grade, have been analyzed and compared between the LR and HR groups as well as within the IOM cohort.

**Figure 5:**
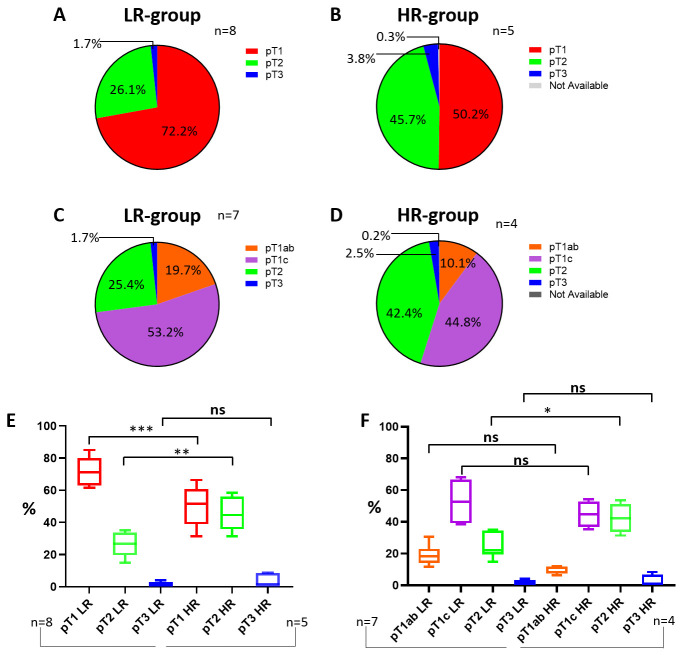
Tumor stage. Pie chart of LR-group tumor size percentages (**A**). Pie chart of HR-group tumor size percentages (**B**). Pie chart of LR-group tumor size percentages with pT1 sub-stratification (**C**). Pie chart of HR-group tumor size percentages with pT1 sub-stratification (**D**). Statistical analysis between LR and HR groups according to tumor stage (**E**) and with sub-stratification of pT1 tumors (**F**). Abbreviations: LR: low-risk; HR: high-risk. The “*“, “**” and "***" symbols represent *p* ≤ 0.05, 0.01 and 0.001; the “ns” symbol represents *p* > 0.05; the “%” symbol on the y-axis refers to aggregated proportions across studies. Calculations are unweighted.

Since the EndoPredict test is intended and developed for primary non-metastatic BCs in women, only female patients with pT1-T3 breast tumors are eligible. As depicted in Figure 5, the LR group displays a higher percentage of stage 1 tumors (pT1) compared to the HR group (72.2% versus 50.2%). On the contrary, the HR group shows almost double (45.7% and 3.8%) values for stage 2 (pT2) and stage 3 (pT3) tumors compared to the LR group (Figure 5A,B). Even inside the pT1 tumor fraction itself, by excluding those studies [16,44,51] that did not report complete sub-stratification of pT1 tumors, the smallest tumors (pT1ab) are more present in the LR group (19.7%) compared to the other one (10.1%) (Figure 5C,D). As a consequence, the major presence of larger tumors is a relevant trait of the HR group, and it justifies the higher number of high-risk subjects.

The statistical analysis, in fact, performed by comparing the LR and HR groups does not highlight any significance when pT1 tumor sub-stratification is accounted for (Figure 5F); however, it arises for both pT1 (as a whole group) and pT2 but not for pT3 tumor stages (Figure 5E).

**Figure 6:**
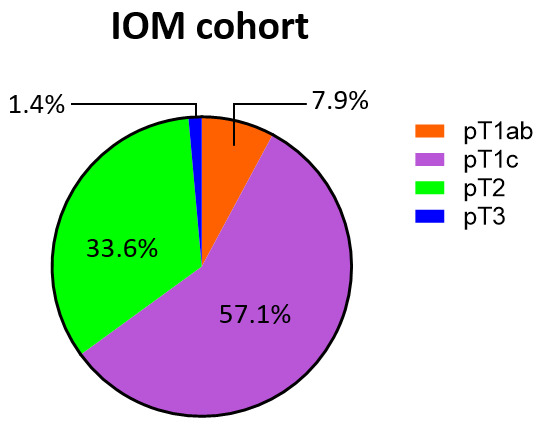
Tumor stage of the IOM cohort. Pie charts of the IOM-cohort tumor size percentages. Abbreviation: IOM: Istituto Oncologico del Mediterraneo.

The IOM cohort displays a profile that appears closer to that of the LR group (Figure 6). However, unlike the previous cohorts, only a small percentage (7.9%) of the pT1 class comprehends pT1ab tumors, which are mostly of the pT1c type (57.1%). In addition, the IOM cohort contains more pT2 tumors as well. The pT1c and pT2 stages are significantly more represented in the IOM cohort (90.7%) than in the LR group (78.3%) and are slightly more numerous compared to the HR group as well (85.1%). This data, along with the evidence that as tumor size increases, the percentage of high-risk EndoPredict results progressively rises (*p* < 0.001, Table 4), consistently suggests that the IOM cohort aligns more closely with a high-risk-profile group.

**Figure 7:**
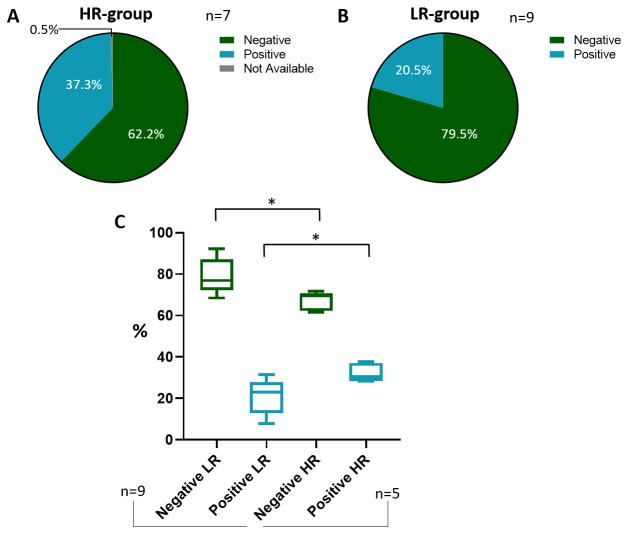
Lymph node status. Pie charts of the lymph node status of the HR group (**A**). Pie chart of the lymph node status composition of the LR group (**B**). Statistical analysis between LR and HR groups according to lymph node status (**C**). Abbreviations: HR: high-risk; LR: low-risk. The “*” symbol represents *p* ≤ 0.05; the “%” symbol on the y-axis refers to aggregated proportions across studies. Calculations are unweighted.

Although most of the tested subjects in both the LR (79.5%) and HR groups (62.2%) are characterized by node-negative BCs, the percentage of node-positive tumors is greater in the HR group (37.3%) compared to the LR group (20.5%) (Figure 7A,B). This difference is not only numerically but also statistically significant: in fact, by excluding two high-risk studies [51,53] whose enrolled subjects are all node-positive or node-negative, the analysis displays a statistically significant difference (Figure 7C).

**Figure 8:**
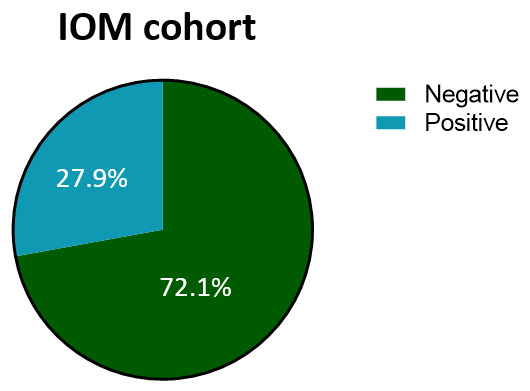
Lymph node status of the IOM cohort. Pie chart illustrating the lymph node status percentages of the IOM cohort. Abbreviation: IOM: Istituto Oncologico del Mediterraneo.

Our cohort, which encompasses a wide number of node-negative BC subjects (72.1%, Figure 8), appears to closely resemble the composition observed in the HR group. Furthermore, when the lymph node status is negative, the percentage of LR/HR scores is almost balanced (Table 5); conversely, the percentage of EP tests considerably shifts towards high-risk-classified scores (*p* < 0.001, Table 5) when the lymph node status is positive.

**Figure 9:**
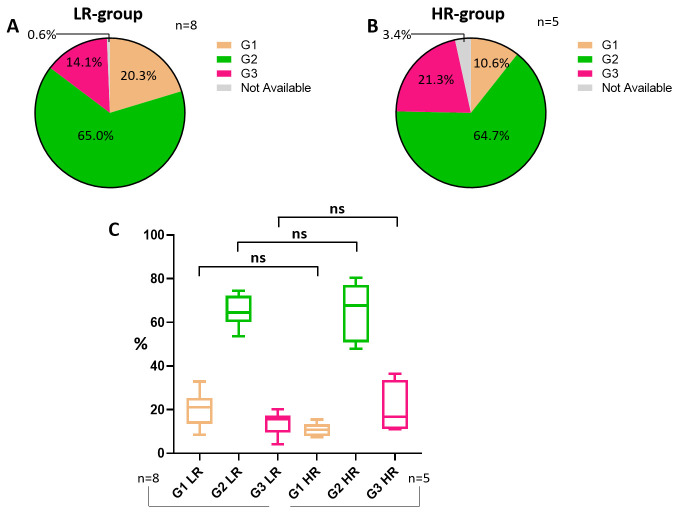
Tumor grade. Pie chart showing tumor grade status in the LR group (**A**). Pie chart illustrating tumor grade status in the HR group (**B**). Statistical analysis comparing tumor grade status between LR and HR groups (**C**). Abbreviations: LR: low-risk; HR: high-risk. The symbol “ns” indicates *p* > 0.05; the “%” symbol on the y-axis refers to aggregated proportions across studies. Calculations are unweighted.

By comparing the LR and HR groups according to tumor grade, which reflects cancer aggressiveness and ranges from 1 to 3, both categories show similar percentages of G2 tumors (65.0% versus 64.7%), while G1 and G3 values are basically reversed (Figure 9A,B). Nevertheless, when comparing both groups regarding the grade parameter, no statistical significance arises (Figure 9C).

**Figure 10:**
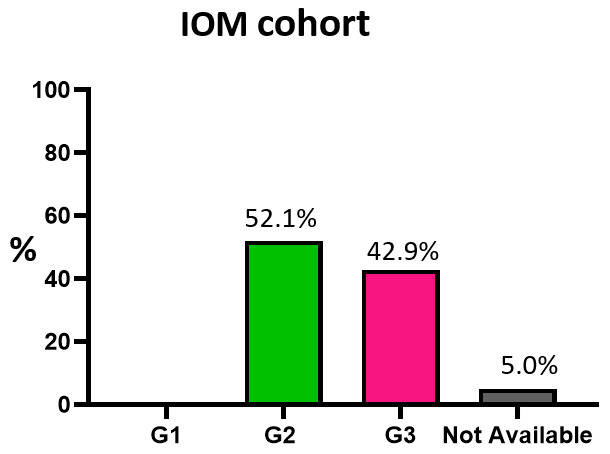
Tumor grade of the IOM cohort. Bar chart depicting the percentages of tumor grade status in the IOM cohort. Abbreviations: IOM: Istituto Oncologico del Mediterraneo. The “%” symbol on the y-axis refers to the whole IOM cohort. Calculations are unweighted.

IOM subjects, unlike those from the literature, display a complete absence of G1 cancers and a larger G3 fraction (Figure 10). In our cohort, while G2 BCs include nearly the same percentages of low- and high-risk results, the G3 fraction has three times the percentage of high-risk subjects compared to low-risk ones (*p* = 0.006, Table 6). Thus, the higher prevalence of G3 cases clearly shifts our cohort towards a high-risk profile.

### 3.3. Molecular and Histological Features: ER, PR, Ki67, Molecular Subtype, and Histology

Several immunophenotypic parameters were evaluated to assess potential associations between these characteristics and the frequency of high-risk cases. However, in the available literature, only a limited number of studies report specific information on the quantitative expression of the estrogen receptor (ER). Only one study, with a predominance of patients with a low EPclin score, provides precise stratification of patients into categories based on different levels of ER expression [16]: a total of 57.1% of patients exhibit high ER expression, while 32.5% have medium ER levels, and 10.4% have low ER expression. The only study with a predominance of patients with a high EPclin score uses a combined stratification strategy based on the expression of both the ER and the progesterone receptor (PR) [44]: a total of 69.6% of patients have a high percentage of hormone-receptor-positive cells, and 29% exhibit low expression, but this information is missing for 1.4% of subjects.

**Figure 11:**
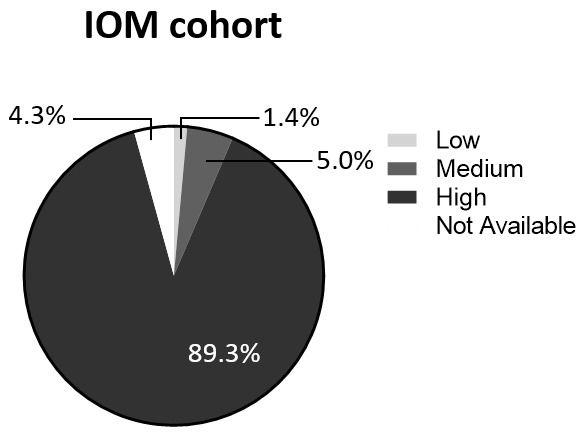
Estrogen receptor expression. Pie chart of IOM-cohort stratification according to ER expression. Abbreviation: IOM: Istituto Oncologico del Mediterraneo.

Overall, the IOM cohort exhibits a different distribution. According to ASCO guidelines [57] only BCs with IHC ER expression above 10% clearly benefit from endocrine therapy, while more controversial data are available for the 1–9% category. From this point of view and exploiting a stratification strategy found in the literature [58], patients were divided into three categories. The percentages of patients with high (above or equal to 90%), medium (70–89%), and low (10–69%) ER expression are 89.3%, 5%, and 1.4%, respectively (Figure 11). The differences in high- and low-risk frequencies across the three ER expression groups were statistically significant (*p* < 0.001, Table 7). Still, since the vast majority of patients in the cohort belongs to the high-ER-expression group, this may affect the robustness of the analysis, and these data need to be interpreted with caution.

**Figure 12:**
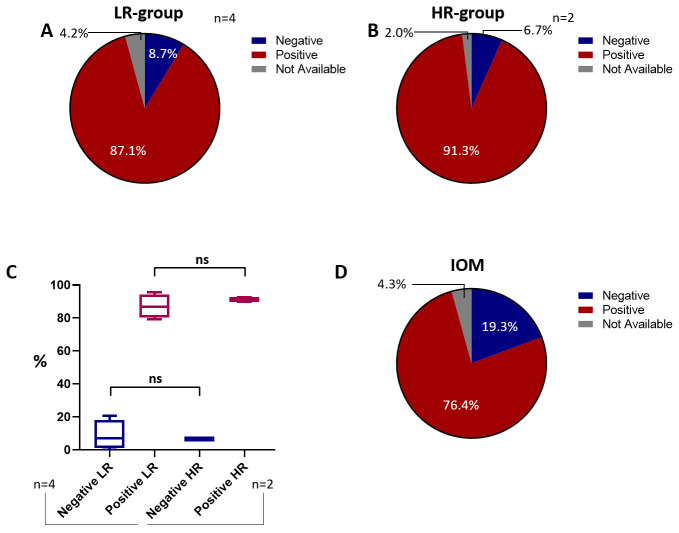
Progesterone receptor expression. Pie charts of the PR expression in the LR group (**A**) and the HR group (**B**). Statistical analysis of LR/HR studies depending on PR expression status (**C**). Pie chart of IOM-cohort PR expression status (**D**). Abbreviations: LR: low-risk; HR: high-risk; PR: progesterone receptor; IOM: Istituto Oncologico del Mediterraneo. The “ns” symbol represents *p* > 0.05; the “%” symbol on the y-axis refers to aggregated proportions across studies. Calculations are unweighted.

A total of six identified studies report details on PR positivity. Of these, four [16,47,49,53] have a higher frequency of low-risk patients, while two [43,55] show a predominance of high-risk individuals (Figure 12A,B). In all the analyzed studies, the predominant fraction consisted of PR-positive patients. The average proportion of PR-negative patients in studies with a predominance of low-risk cases was 8.7%, while in studies with a predominance of high-risk cases, it was 6.7%. The differences in the frequency of PR-positive receptors between the two groups of studies were not statistically significant (Figure 12C).

Note that all previous statistical analyses comparing both LR and HR groups on the basis of a certain parameter and their interpretations are hypothesis-generating and not confirmatory due to the descriptive/scoping nature of our review.

Similarly to ER expression status, IOM patients were firstly stratified on the basis of the cut-off value (20%) reported in the guidelines of the Italian Ministry of Health and based on recommendations from the Italian Society of Medical Oncology (AIOM) [6]. They show a higher proportion of PR-negative BCs compared to both previous groups of studies (19.3% Figure 12D). Among PR-negative patients, 67% exhibit a high-risk profile, while the remaining 33% are classified as low-risk (Table 8). Similarly, 65% of PR-positive patients are at a high risk, while 35% are at a low risk. The differences in high- and low-risk frequencies between the patient groups are not statistically significant (*p* = 0.88).

**Figure 13:**
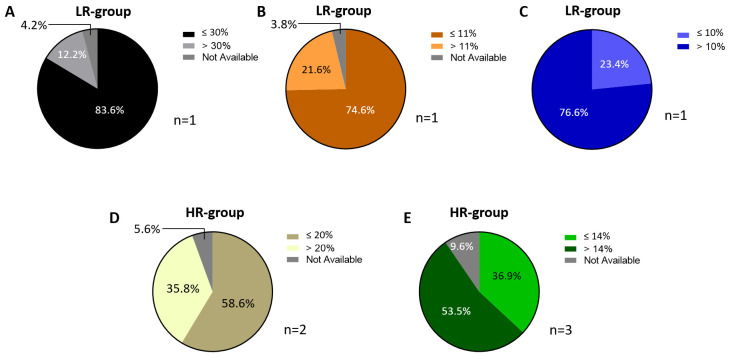
Ki-67. Pie charts of BC patients from LR studies using 30% (**A**), 11% (**B**) and 10% (**C**) as the Ki-67 cut-off values. Pie charts of BC patients from HR studies that classify them by considering 20% (**D**) and 14% (**E**) as the cut-off values. Abbreviations: BC: breast cancer; LR: low-risk; HR: high-risk.

Few studies report detailed information on Ki-67 expression levels: three [16,42,47] and five articles [43,48,50,52,55] with a predominance of low-risk and high-risk patients, respectively. Moreover, since the Ki-67 thresholds used to stratify patients vary across studies, data aggregation and a comparison with our data are challenging. One study [42] employs a Ki-67 threshold of 30% to classify patients, identifying 83.6% as having low Ki-67 expression and 12.2% as having high Ki-67 expression, with 4.2% of cases lacking this information (Figure 13A). Two additional studies use lower, but comparable, thresholds: Filipits et al. sets their threshold at 11%, while Jahn et al. uses a threshold of 10%. The cohort in Filipits et al.’s study shows a distribution similar to that of Constantinidou et al., with 74.6% of patients classified as having low Ki-67 expression and 21.6% as having high Ki-67 expression, with 3.8% missing data (Figure 13B). However, in Jahn et al.’s study, where the threshold is set at 10%, the distribution was reversed, with only 23.4% of patients having Ki-67 levels ≤ 10% and 76.6% exhibiting levels above it (Figure 13C). A similar pattern is observed in studies focusing on high-risk patient groups. In two studies [48,55], a Ki-67 threshold of 20% is applied, and the proportion of patients classified as having high Ki-67 expression is substantially larger, with 58.6% falling into the low-Ki-67 category and 35.8% into the high-Ki-67 category (Figure 13D). Likewise, in studies that use a threshold of 14% [43,50,52], high Ki-67 expression is more prevalent than low Ki-67 expression. Specifically, 53.5% of patients exhibit Ki-67 levels above 14%, while 36.9% have Ki-67 levels at or below this threshold (Figure 13E). These findings highlight the inconsistency in Ki-67 stratification criteria across studies, which complicates direct comparisons and meta-analyses. The observed trend of higher Ki-67 expression being more frequent in high-risk patient groups may suggest a potential association between Ki-67 levels and patient risk stratification. However, it is important to underline the narrative interpretation of this trend because, as emphasized before, the wide nature of selected cut-off values among analyzed studies makes it difficult to draw inferential statements across studies. Nevertheless, further investigation about this trend would be reasonable.

**Figure 14:**
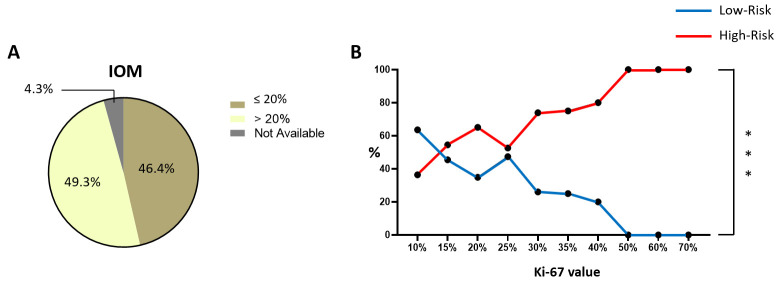
Ki-67 expression of the IOM cohort. Pie chart of IOM-cohort percentages based on 20% as the Ki-67 cut-off value (**A**). Graphic representation of the statistical analysis of Ki-67 expression in IOM LR/HR cases (**B**). Abbreviations: IOM: Istituto Oncologico del Mediterraneo; LR: low-risk; HR: high-risk. The “***” symbol represents *p* ≤ 0.001; the “%” symbol on the y-axis refers to a subgroup of the IOM cohort (information that was not available was excluded). Calculations are unweighted.

According to the guidelines of the Italian Ministry of Health and based on recommendations from AIOM [6], a Ki-67 threshold of 20% has been established as a significant parameter for risk classification. Consequently, the IOM patients were first stratified according to this criterion. Notably, the majority of patients in the cohort exhibited a Ki-67 value equal to 20% (Figure 14A and Table 9). This particular enrichment may be due to the fact that surpassing this threshold is one of the five criteria used to classify a patient as having an intermediate or high risk. Moreover, the ratio of high-to-low Ki67 patients in the IOM cohort (49.3% and 46.4%, respectively) is comparable to that observed in studies featuring a predominance of high-risk patients (Figure 13D,E and Figure 14A). Although the limited number of available studies and the variability in Ki-67 thresholds prevent a comprehensive evaluation of these similarities, these findings suggest that the IOM cohort may be classified as a high-risk population.

Additionally, we decided to further stratify our cohort by exploiting every single value of Ki-67, from 10% to 70%. As depicted in Figure 14B, we, thus, observed the clear discrimination between LR- and HR-classified patients when Ki-67 is above 25%. In fact, when its value is between 10% and 25%, the probability of being classified as a high or low risk of recurrence varies, as it probably depends on other factors. On the contrary, when the Ki-67 value overcomes 25%, the probability of having a high-risk result progressively increases as Ki-67 becomes higher.

The statistical analysis performed additionally supports this hypothesis (*p* = 0.0001, Table 9). Further analysis with larger and more standardized cohorts could offer greater clarity on this hypothesis.

EndoPredict is used in hormone-receptor-positive disease and, under standard conditions, is primarily administered to patients with the Luminal A or Luminal B phenotype. In the analyzed studies, information on surrogate molecular subtypes is never explicitly reported, preventing a systematic assessment of its potential implications in risk evaluation.

**Figure 15:**
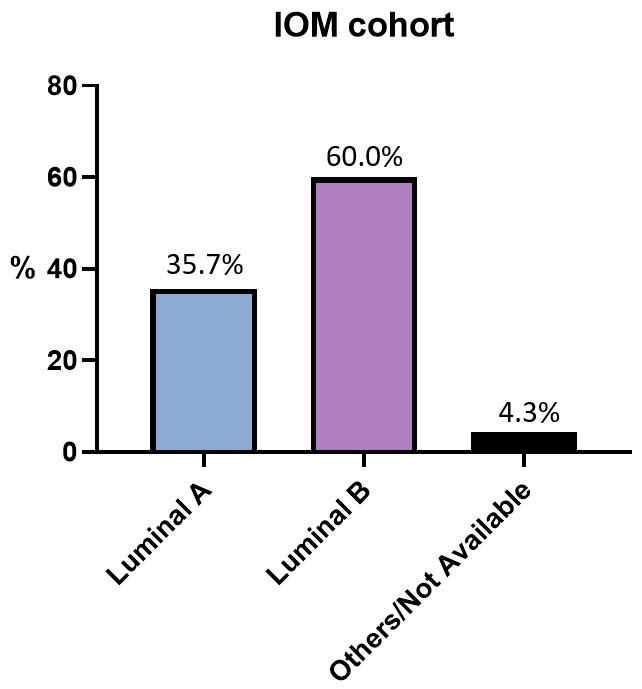
Surrogate intrinsic subtypes. Bar chart of IOM-cohort surrogate intrinsic subtypes. The “%” symbol on the y-axis refers to the whole IOM cohort. Abbreviation: IOM: Istituto Oncologico del Mediterraneo. Calculations are unweighted.

In the IOM case series (Figure 15), 60% of patients have a Luminal B phenotype, 35.7% have a Luminal A phenotype, while in 4.3% of cases, this information was not retrieved. Among the 50 patients with a Luminal A phenotype, 20 (40%) were classified as low-risk according to the EndoPredict test, while 30 (60%) were classified as high-risk (Table 10). Among the 84 patients with a Luminal B phenotype, 26 (31%) were classified as low-risk, while 58 (69%) were classified as high-risk. The slight differences observed in risk distribution between the two phenotypic groups were not statistically significant (*p* = 0.24). This finding should be interpreted in light of the clinical selection criteria for EndoPredict testing, which is performed on a subset of patients with intermediate clinical risk rather than on the entire Luminal A or B population. The EPclin risk classification depends, in fact, on the gene expression signature, which also adjusts for tumor size and nodal status, not on the luminal phenotype itself.

The histology of tumors was analyzed in studies where this information was available: five [45,46,47,49,53] and three studies [50,51,55] with a prevalence of low-risk and high-risk subjects, respectively. Considering only the invasive subtype, in the group of studies with a predominance of low-risk patients, the average percentage of ductal carcinomas is 78.53%, while the percentage of lobular carcinomas is 13.09%. Additionally, an average of 8.48% of patients in this group have no available information or exhibit a different phenotype (Table S7). In the group of studies with a predominance of high-risk patients, the average percentage of ductal carcinomas is 82.66%, while the percentage of lobular carcinomas is 5.78%. Additionally, an average of 11.55% of patients in this group have no available information or exhibit a different phenotype (Figure S3A,B, Table S7).

In the IOM cohort, the percentage of ductal and lobular carcinomas is 85% and 7.9% respectively, while 7.1% of patients have no available information or exhibit a different phenotype (Figures S3C and S4A–C). Since the majority of patients (119) have an invasive ductal histotype, the differences observed between ductal and lobular samples are not statistically significant (*p* = 0.28, Table S8).

To further assess the independent contribution of clinical and biological factors, we performed multivariate logistic regression that includes age, number of pregnancies, tumor size, nodal status, histological grade, and Ki-67 (Table 11). Tumor size, nodal involvement, and the proliferative index remained independent predictors of a high-risk classification according to EPclin. Using Ki-67 as a continuous variable, pT1c (OR 7.12; 95% CI 1.25–40.60; *p* = 0.027), pT2 (OR 38.99; 95% CI 5.39–281.95; *p* = 0.0003), and node-positive disease (OR 5.24; 95% CI 1.48–18.57; *p* = 0.010) were significantly associated with a high risk. Ki-67 was independently associated with a high risk as well (OR 1.11 per 1% increase; 95% CI 1.03–1.19; *p* = 0.006). The model’s AUC was 0.846.

In an alternative specification using Ki-67 ≥ 20% (vs. <20%), pT2 (OR 27.08; 95% CI 4.12–178.20; *p* = 0.0006), node-positive disease (OR 6.84; 95% CI 1.71–27.29; *p* = 0.006), and Ki-67 ≥ 20% (OR 4.79; 95% CI 1.06–21.65; *p* = 0.042) were significantly associated with a high risk, whereas pT1c showed a positive but non-significant trend (OR 3.95; 95% CI 0.81–19.24; *p* = 0.089). Age showed a borderline inverse association with high-risk classification (OR 0.95 per year; 95% CI 0.91–1.00; *p* = 0.044), while the number of pregnancies was not significant. The model’s AUC was 0.824. The complete results of the multivariable logistic regression models are reported in Supplementary Table S4.

## 4. Discussion

The analysis of the relevant scientific literature highlights the significant variability in the reported frequencies of high-risk (HR) and low-risk (LR) classifications across different studies evaluating EndoPredict (EP) in the clinical context of breast cancer (BC), despite the use of the same genomic assay. Some clinical settings seem to have a higher proportion of high-risk individuals compared to others, likely due to population-specific factors.

Our analysis, in fact, suggests that these discordant distributions of HR and LR patients may be effectively attributed to intrinsic clinical and biological differences within the involved patient cohorts. As expected, tumor size and lymph node status remain primary determinants of EP risk classification. Both parameters are differently represented in both categories: larger cancers and greater lymph node involvement have been found in the HR group compared to the LR one. Furthermore, we observed a higher percentage of G3 tumors, and a lower presence of G1 ones in the HR group. Although the chosen Ki-67 cut-off values make it challenging to draw clear conclusions, the LR group seems to include BCs with lower Ki-67 levels than the HR group. This observation consistently agrees with previous traits, depicting a worse and advanced BC status among HR subjects. It is important to note that these observations should not be interpreted as novel biological determinants of genomic risk, but rather as factors influencing the distribution of EPclin risk categories across different patient populations. Conversely, parameters such as age, menopausal status, ER/PR expression, and histology did not demonstrate significant discriminatory power due to the poor quality of the retrieved data and the variability across the analyzed studies.

The IOM cohort, which predominantly includes HR-classified cases, displayed a tumor profile characterized by larger sizes, higher histological grades, greater lymph node involvement, and higher proliferative activity. This finding aligns with the hypothesis that different hospital populations contribute to the observed variability in EP risk distributions. In addition to the already cited clinicopathological features, we evaluated the potential association between EP frequencies and other parameters in our population.

Focusing on age, we report the indication of an unexpected higher presence of older subjects in the HR group compared to the LR one in this study. Other studies, however, should deepen their analysis if this indication is due to population-specific factors inside our cohort or represents a further putative association between age and BC risk, thereby adding to the evidence that BC incidence increases with age. It could be eventually justified by physiological changes associated with post-menopausal status [59]. Both the chosen cut-off value of “50 years old”, which belongs to the natural menopause range of 45 to 55 years worldwide, and the marked predominance of post-menopausal subjects (62.1%) compared to pre-menopausal ones (27.9%) might support this hypothesis.

Regarding estrogen receptor (ER) expression, we noticed a statistically significant association between higher ER expression status and BC recurrence risk. However, the low quality of our data, which mainly encompass BCs strongly positive for ERs, may have introduced bias in our analysis. Thus, this result needs to be considered with caution.

On the contrary, age of menarche, menopausal status, BMI, progesterone receptor expression, molecular subtype, and histology did not reveal significant insights. Instead, a higher number of pregnancies was considerably associated with a lower frequency of high-risk classifications, which could be explained by pregnancy-induced anatomic, hormone, genetic, and epigenetic modifications. Changes in hormone levels during pregnancy would promote the differentiation of breast epithelial cells and decrease mammary stem cells, thus limiting the pool of potentially tumorigenic events of the mammary gland [60]. Along with an overall improved hormone-mediated responsiveness of the mammary gland, Barton et al. reported a pregnancy-induced “genomic signature” that would comprise genetic and epigenetic modifications, thereby resulting in reduced susceptibility of the epithelial cells to carcinogenesis [61]. Our data may be consistent with epidemiological evidence suggesting that reproductive history may influence breast cancer biology and its evolution as a high-/low-risk event [62]; nevertheless, our results should be cautiously interpreted given the exploratory nature of the analysis.

Furthermore, the multivariate analysis performed in our cohort showed that classical clinical parameters such as tumor size, nodal involvement, and proliferative activity were associated with a higher frequency of patients being classified as high-risk by EPclin. This finding is not unexpected, since these variables are either directly included in the algorithm or closely related to its molecular components. Therefore, the purpose of this analysis was not to identify new predictors of EPclin risk but to explore how differences in clinicopathological characteristics across cohorts may contribute to the variability in the observed distribution of EPclin classifications. Ki-67, in particular, was consistently associated with the frequency of high-risk classification, both when analyzed as a continuous variable and when dichotomized at the conventional 20% cut-off, in line with the St. Gallen consensus for luminal breast cancer subtypes. Age, unlike what was observed in the univariate analysis, displayed a borderline inverse association with high-risk classification, consistent with the observation that younger patients are more often represented in the high-risk group due to a more aggressive disease. In contrast, reproductive variables such as pregnancy, as suggested by the univariate analysis, did not retain significance once tumor-related factors were accounted for. Overall, these results indicate that some clinical and demographic characteristics are associated with the distribution of patients across EPclin risk groups and may contribute to the heterogeneity observed when comparing different study populations.

Unlike first-generation genomic assays (Oncotype DX^®^, the Breast Cancer Index and MammaPrint^®^), which solely rely on gene expression profiles, second-generation tests like EndoPredict^®^ and Prosigna^®^ incorporate clinical variables, allowing for a more tailored risk assessment. Although their clinical validity has been supported by several clinical trials and they have even been recommended for specific applications by American Society of Clinical Oncology (ASCO) guidelines [63], EndoPredict seems to outperform the others in some aspects [22,36,41,45,47]. Specifically, beyond its practical implementation “in house” in clinical routine compared to the mandatory external centralization of the first-generation tests, it covers a broader predictive range of early and late distant recurrence risk. Moreover, it is the only one, along with Oncotype DX, to provide indications on the benefit of chemotherapy [64]. Also, the integration of tumor size and nodal status with molecular data strengthens its prognostic and predictive value, leading to better discrimination of the heterogeneity observed across different patient populations [24,32]. With the aim of emphasizing the importance of integrating clinical parameters for a better evaluation of BC patients, it has recently been reported that the Oncotype DX recurrence score increases concordance in adjuvant chemotherapy recommendations when evaluated in combination with tumor grade, size, and patient age [65]. Thus, the routinary application of these genomic assays has prompted both improvements regarding the interpretation of their results (e.g., eliminating the ambiguity of an intermediate-risk category that originally characterized Oncotype DX) and efforts to combine them with clinical parameters. Also, increased comprehension will allow for a better selection of candidate subjects and, consequently, will guide more patients to the proper therapeutic regimen.

## 5. Conclusions

In conclusion, the observed variability underscores the importance of prognostic tests that integrate both molecular signatures and clinical parameters for a more comprehensive risk stratification. Given the considerable diversity in breast cancer presentation, these integrative models are likely to be more effective in personalizing treatment decisions, ensuring that high-risk patients receive appropriate adjuvant therapy while avoiding overtreatment in low-risk cases. Future research should further explore the impact of patient population differences on genomic test outcomes and assess how emerging biomarkers could further refine risk stratification in clinical practice.

## Figures and Tables

**Table 1:** IOM cohort patients’ clinical characteristics.

Clinical Parameter	Characteristic	Number (%)
EPclin score	Low-Risk	48 (34%)
	High-Risk	92 (66%)
Age	≤50 y.o.	39 (28%)
	>50 y.o.	101 (72%)
Age of menarche	≤12 y.o.	87 (62%)
	>12 y.o.	46 (33%)
	Not Available	7 (5%)
Menopausal status	Pre-menopausal	39 (28%)
	Post-menopausal	87 (62%)
	Not Available	14 (10%)
Number of pregnancies	≤2	102 (73%)
	>2	28 (20%)
	Not Available	10 (7%)
Body mass index (BMI)	Underweight	3 (2%)
	Normal	41 (29%)
	Overweight	30 (21%)
	Obese	12 (9%)
	Not Available	54 (39%)
Tumor stage	pT1ab	11 (8%)
	pT1c	80 (57%)
	pT2	47 (34%)
	pT3	2 (1%)
Lymph node status	Negative	101 (72%)
	Positive	39 (28%)
Grade	1	0 (0%)
	2	73 (52%)
	3	60 (43%)
	Not Available	7 (5%)
Estrogen receptor (ER) status	Low (10–69%)	2 (1%)
	Medium (70–89%)	7 (5%)
	High (≥90%)	125 (90%)
	Not Available	6 (4%)
Progesterone receptor (PR) status	Negative (<20%)	27 (19%)
	Positive (≥20%)	107 (77%)
	Not Available	6 (4%)
Ki-67	≤20%	65 (47%)
	>20%	69 (49%)
	Not Available	6 (4%)
Molecular subtype	Luminal A	50 (36%)
	Luminal B	84 (60%)
	Other/Not Available	6 (4%)
Histological subtype	Invasive Ductal (No Special Type)	119 (85%)
	Invasive Lobular	11 (8%)
	Other/Not Available	10 (7%)

**Table 2:** Table presenting the numbers and relative percentages of LR and HR patients across all considered categories based on the age of menarche parameter. Abbreviations: LR: low-risk; HR: high-risk.

Age (y/o)	Low-Risk (%)	High-Risk (%)
9	3 (100%)	0 (0%)
10	2 (33%)	4 (67%)
11	15 (40%)	23 (60%)
12	13 (33%)	27 (67%)
13	9 (31%)	20 (69%)
14	4 (31%)	9 (69%)
15	0 (0%)	3 (100%)
16	1 (100%)	0 (0%)
Not Available	1 (14%)	6 (86%)

**Table 3:** Table displaying the numbers and relative percentages of LR/HR patients among the different recorded numbers of pregnancies. Abbreviations: LR: low-risk; HR: high-risk.

Number of Pregnancies	Low-Risk (%)	High-Risk (%)
0	5 (21%)	19 (79%)
1	8 (31%)	18 (69%)
2	20 (39%)	32 (61%)
3	9 (38%)	15 (62%)
4	2 (50%)	2 (50%)
Not Available	4 (40%)	6 (60%)

**Table 4:** Table reporting the number and the relative percentages of LR/HR subjects in all defined categories. Abbreviations: LR: low-risk; HR: high-risk.

Tumor Stage	Low-Risk (%)	High-Risk (%)
pT1ab	8 (73%)	3 (27%)
pT1c	34 (43%)	46 (57%)
pT2	6 (13%)	41 (87%)
pT3	0 (0%)	2 (100%)

**Table 5:** Table presenting the number and relative percentages of LR/HR subjects across all defined categories Abbreviations: LR: low-risk; HR: high-risk.

Lymph Node Status	Low-Risk (%)	High-Risk (%)
Negative	42 (42%)	59 (58%)
Positive	6 (15%)	33 (85%)

**Table 6:** Table presenting the numbers and relative percentages of LR/HR subjects across all defined categories. Abbreviations: LR: low-risk; HR: high-risk.

Grade Status	Low-Risk (%)	High-Risk (%)
G1	0 (0%)	0 (0%)
G2	31 (42%)	42 (58%)
G3	14 (23%)	46 (77%)
Not Available	3 (43%)	4 (57%)

**Table 7:** Table displaying the numbers and relative percentages of LR/HR subjects across all defined categories. Abbreviations: ER: estrogen receptor; LR: low-risk; HR: high-risk.

ER Expression Status	Low-Risk (%)	High-Risk (%)
Low	0 (0%)	2 (100%)
Medium	4 (57%)	3 (43%)
High	42 (34%)	83 (66%)
Not Available	2 (33%)	4 (67%)

**Table 8:** Table reporting the numbers and relative percentages of LR/HR subjects among PR Expression status categories. Abbreviations: LR: low-risk; HR: high-risk.

PR Expression Status	Low-Risk (%)	High-Risk (%)
Negative	9 (33%)	18 (67%)
Positive	37 (35%)	70 (65%)
Not Available	2 (33%)	4 (67%)

**Table 9:** Table reporting the numbers and relative percentages of LR/HR subjects in all defined Ki-67 categories. Abbreviations: LR: low-risk; HR: high-risk.

Ki-67 Value	Low-Risk (%)	High-Risk (%)
10%	7 (64%)	4 (36%)
15%	5 (46%)	6 (54%)
20%	15 (35%)	28 (65%)
25%	9 (47%)	10 (53%)
30%	6 (26%)	17 (74%)
35%	1 (25%)	3 (75%)
40%	3 (20%)	12 (80%)
50%	0 (0%)	5 (100%)
60%	0 (0%)	2 (100%)
70%	0 (0%)	1 (100%)
Not Available	2 (33%)	4 (67%)

**Table 10:** Table reporting the numbers and relative percentages of surrogate intrinsic subtypes in all defined categories.

Intrinsic Subtype Status	Low-Risk (%)	High-Risk (%)
Luminal A	20 (40%)	30 (60%)
Luminal B	26 (31%)	58 (69%)
Others/Not Available	2 (30%)	4 (70%)

**Table 11:** Multivariate logistic regression analysis. Table reporting the results of the multivariate logistic regression analysis that included age, number of pregnancies, tumor size, nodal status, histological grade, and Ki-67. Abbreviations: OR: odds ratio; CI: confidence interval.

Ki-67 as Continuous Variable			Ki-67 with 20% Threshold		
Variable	OR (95% CI)	*p*-Value	Variable	OR (95% CI)	*p*-Value
Ki-67 (1% increase)	1.11 (1.03–1.19)	0.006	Ki-67 ≥ 20% vs. <20%	4.79 (1.06–21.65)	0.042
Tumor size: pT1c vs. pT1ab	7.12 (1.25–40.60)	0.027	Tumor size: pT1c vs. pT1ab	3.95 (0.81–19.24)	0.089
Tumor size: pT2 vs. pT1ab	38.99 (5.39–281.95)	0.0003	Tumor size: pT2 vs. pT1ab	27.08 (4.12–178.20)	0.0006
Nodal Status: positive vs. negative	5.24 (1.48–18.57)	0.010	Nodal Status: positive vs. negative	6.84 (1.71–27.29)	0.006
Age (per year)	0.96 (0.92–1.01)	0.083	Age (per year)	0.95 (0.91–1.00)	0.044
Pregnancies (per unit)	0.93 (0.57–1.51)	0.768	Pregnancies (per unit)	0.86 (0.55–1.37)	0.537

## Data Availability

All data are available in aggregated form in the Supplementary Materials.

## Supplementary Materials

The following supporting information can be downloaded at: https://www.mdpi.com/article/10.3390/cancers18060951/s1, Table S1: List of excluded studies and primary reasons for exclusion; Table S2: List of included studies and key inclusion criteria; Table S3: Descriptive sensitivity analysis of cohort grouping criteria (Δ > 5% vs. Δ > 10%) and characterization of near-balanced cohorts; Table S4: Full multivariable logistic regression results; Table S5: Table of numbers and percentages of LR/HR cases for the three indicated categories; Table S6: Table reporting the numbers and relative percentages of LR/HR-subjects in all defined categories. Abbreviations: LR: Low-Risk; HR: High-Risk; Table S7: Table reporting LR- and HR-groups percentages and ranges for histological subtypes. Abbreviations: NST: non-special type; LR: Low-Risk; HR: High-Risk; Table S8: Table reporting the relative percentages of histological subtypes in all defined categories. Abbreviation: NST: non-special type. Figure S1: Menopausal status; Figure S2: Body Mass Index; Figure S3: Histological subtypes; Figure S4: Histological subtypes of the IOM cohort.

## Abbreviations

AIOM	Associazione Italiana di Oncologia Medica
ASCO	American Society of Clinical Oncology
AUC	Area Under the Curve
BC	Breast Cancer
BMI	Body Mass Index
CI	Confidence Interval
EP	EndoPredict
EPclin	EndoPredict Clinical
ER	Estrogen Receptor
HER2	Human Epidermal growth factor Receptor 2
IHC	Immunohistochemistry
IOM	Istituto Oncologico del Mediterraneo
HR	Hormone Receptor
HR	High-Risk
LR	Low-Risk
OR	Odds Ratio
PCR	Polymerase Chain Reaction
PR	Progesterone Receptor
ROC	Receiver Operating Characteristic
VIFs	Variance Inflation Factors
