# Supplementary material for: The Impact of Clinical and Demographic Factors on High-Risk Patient Classification Frequencies by the EndoPredict Test: A Review and Single-Site Study
Source: Cancers (Basel). 2026 Mar 14;18(6):951. doi: 10.3390/cancers18060951 (PMC13026031; doi:10.3390/cancers18060951)
Supplement: Supplementary file 1 [file cancers-18-00951-s001.zip › cancers-4166877-supplementary.pdf]

Reference n.	First author (Year)	Stage of Exclusion	Primary Reason for exclusion	Notes
19	Bertucci 2014	Initial screening	Near-balanced LR/HR distribution (Δ <5%)	51% LR vs 49% HR
20	Fitzal F (2015)	Initial screening	Near-balanced LR/HR distribution (Δ <5%)	48% LR vs 52% HR
21	Sandoval (2024)	Initial screening	Near-balanced LR/HR distribution (Δ <5%)	52% LR vs 48% HR
22	Mokbel K (2017)	Initial screening	Balanced LR/HR distribution (Δ 0%)	50% LR vs 50% HR
23	Peláez-García A (2017)	Initial screening	Balanced LR/HR distribution (Δ 0%)	50% LR vs 50% HR
24	Mokbel K (2018)	Initial screening	Balanced LR/HR distribution (Δ 0%)	50% LR vs 50% HR
25	Schmitt (2023)	Initial screening	Near-balanced LR/HR distribution (Δ <5%)	49% LR vs 51% HR
17	Dubsky P (2013)	Post-grouping exclusion	Overlapping cohort	Cohort already included in Filipits M (2011)
18	Filipits (2019)	Post-grouping exclusion	Overlapping cohort	Cohort already included in Filipits M (2011)
26	Sestak I (2018)	Post-grouping exclusion	Overlapping cohort	Cohort already included in Buus R (2016)
27	Sestak I (2020)	Post-grouping exclusion	Overlapping cohort	Cohort already included in Filipits M (2011) and in Buus R (2016)
28	Vazquez-Juarez D (2022)	Post-grouping exclusion	Overlapping cohort	Cohort already included in Villarreal-Garza C (2020)
29	Pelliccia C (2021)	Post-grouping exclusion	Missing clinicopathological characteristics and enrolment of male subjects (outside EndoPredict indication)	
30	Di Lascio (2022)	Post-grouping exclusion	Missing clinicopathological characteristics	
31	Martin M (2014)	Post-grouping exclusion	Enrolment of ER-negative population (outside EndoPredict indication)	
32	Martin M (2016)	Post-grouping exclusion	Enrolment of ER-negative population (outside EndoPredict indication)	
33	Bösl A (2017)	Post-grouping exclusion	Enrolment of ER-negative population (outside EndoPredict indication)	
34	Almstedt K (2020)	Post-grouping exclusion	Enrolment of ER-negative population (outside EndoPredict indication)	
35	Penault-Llorca F (2024)	Post-grouping exclusion	Enrolment of ER-negative population (outside EndoPredict indication)	
36	Varga Z (2013)	Post-grouping exclusion	Enrolment of HER2-positive population (outside EndoPredict indication)	

37	Noske A (2020)	Post-grouping exclusion	Enrolment of male subjects (outside EndoPredict indication)	
38	Klein E (2024)	Post-grouping exclusion	Enrolment of male subjects (outside EndoPredict indication)	
39	Chow (2018)	Post-grouping exclusion	Presence of subjects with unclassified EP and EPclin score	

Table S1: List of excluded studies and primary reasons for exclusion. Abbreviations: LR: Low-Risk; HR: High-Risk.

Reference n.	First author (Year)	Inclusion criteria	Notes
16	Filipits M (2011)	Not balanced LR/HR distribution (Δ > 5%) and cohort suitable for EndoPredict test	63% LR vs 37% HR
40	Brufsky A (2022)	Not balanced LR/HR distribution (Δ > 5%) and cohort suitable for EndoPredict test	60% LR vs 40% HR
41	Buus R (2016)	Not balanced LR/HR distribution (Δ > 5%) and cohort suitable for EndoPredict test	59% LR vs 41% HR
42	Constantinidou A (2022)	Not balanced LR/HR distribution (Δ > 5%) and cohort suitable for EndoPredict test	65% LR vs 35% HR
43	Dinh P (2022)	Not balanced LR/HR distribution (Δ > 5%) and cohort suitable for EndoPredict test	44% LR vs 56% HR
44	Dubsky PC (2020)	Not balanced LR/HR distribution (Δ > 5%) and cohort suitable for EndoPredict test	24% LR vs 76% HR
45	Ettl J (2017)	Not balanced LR/HR distribution (Δ > 5%) and cohort suitable for EndoPredict test	63% LR vs 37% HR
46	Ettl J (2020)	Not balanced LR/HR distribution (Δ > 5%) and cohort suitable for EndoPredict test	64% LR vs 36% HR
47	Jahn SW (2020)	Not balanced LR/HR distribution (Δ > 5%) and cohort suitable for EndoPredict test	55% LR vs 45% HR
48	Jank P (2022)	Not balanced LR/HR distribution (Δ > 5%) and cohort suitable for EndoPredict test	38% LR vs 62% HR
49	Jung W (2022)	Not balanced LR/HR distribution (Δ > 5%) and cohort suitable for EndoPredict test	74% LR vs 26% HR
50	Lehmann-Che J (2021)	Not balanced LR/HR distribution (Δ > 5%) and cohort suitable for EndoPredict test	44% LR vs 56% HR
51	LaCroix-Triki M (2019)	Not balanced LR/HR distribution (Δ > 5%) and cohort suitable for EndoPredict test	33% LR vs 67% HR
52	Müller BM (2013)	Not balanced LR/HR distribution (Δ > 5%) and cohort suitable for EndoPredict test	46% LR vs 54% HR
53	Penault-Llorca F (2020)	Not balanced LR/HR distribution (Δ > 5%) and cohort suitable for EndoPredict test	68% LR vs 32% HR

54	Penault-Llorca F (2022)	Not balanced LR/HR distribution (Δ > 5%) and cohort suitable for EndoPredict test	14% LR vs 86% HR
55	Villarreal-Garza C (2020)	Not balanced LR/HR distribution (Δ > 5%) and cohort suitable for EndoPredict test	46% LR vs 54% HR

Table S2: List of included studies and key inclusion criteria. Abbreviations: LR: Low-Risk; HR: High-Risk.

<table>
<tr><th></th><th colspan="2">Δ>5% (original Threshold)</th><th colspan="2">Δ>10% (modified Threshold)</th><th>Balanced cohorts (Δ<5%)</th></tr>
<tr><th></th><th>LR-predominant (9 studies)</th><th>HR-predominant (8 studies)</th><th>LR-predominant (8 studies)</th><th>HR-predominant (6 studies)</th><th>5 studies</th></tr>
<tr><td>Tumor size pT1ab (%)</td><td>19,68</td><td>10,13</td><td>21,01</td><td>9,32</td><td>16,39</td></tr>
<tr><td>Tumor size pT1c (%)</td><td>53,24</td><td>44,80</td><td>50,77</td><td>51,56</td><td>46,67</td></tr>
<tr><td>Tumor size pT1 (%)</td><td>72,15</td><td>50,21</td><td>71,06</td><td>51,04</td><td>64,95</td></tr>
<tr><td>Tumor size pT2 (%)</td><td>25,35</td><td>42,43</td><td>26,99</td><td>44,86</td><td>31,42</td></tr>
<tr><td>Tumor size pT3 (%)</td><td>1,72</td><td>2,52</td><td>2,28</td><td>5,23</td><td>4,85</td></tr>
<tr><td>Node-negative (%)</td><td>79,47</td><td>62,20</td><td>80,49</td><td>60,40</td><td>72,53</td></tr>
<tr><td>Node-positive (%)</td><td>20,53</td><td>37,33</td><td>19,51</td><td>39,06</td><td>27,47</td></tr>
<tr><td>Tumor grade G1 (%)</td><td>20,34</td><td>10,64</td><td>21,57</td><td>10,43</td><td>12,94</td></tr>
<tr><td>Tumor grade G2 (%)</td><td>65,01</td><td>64,67</td><td>64,57</td><td>60,65</td><td>68,15</td></tr>
<tr><td>Tumor grade G3 (%)</td><td>14,06</td><td>21,26</td><td>13,18</td><td>26,15</td><td>17,08</td></tr>
<tr><td>ER expression Low (%)</td><td>10,4</td><td rowspan="2">29,03</td><td>10,4</td><td rowspan="2">29,03</td><td>7,48</td></tr>
<tr><td>ER expression Medium (%)</td><td>32,49</td><td>32,49</td><td>30,29</td></tr>
<tr><td>ER expression High (%)</td><td>57,11</td><td>69,59</td><td>57,11</td><td>69,59</td><td>62,24</td></tr>
<tr><td>PgR expression Negative (%)</td><td>8,67</td><td>6,68</td><td>8,36</td><td>7,30</td><td>12,77</td></tr>
<tr><td>PgR expression Positive (%)</td><td>87,09</td><td>91,30</td><td>85,97</td><td>92,70</td><td>87,23</td></tr>
<tr><td>Premenopausal status (%)</td><td>33,33</td><td>67,38</td><td>33,33</td><td>34,76</td><td>0</td></tr>
<tr><td>Postmenopausal status (%)</td><td>66,67</td><td>32,62</td><td>66,67</td><td>65,24</td><td>100</td></tr>
<tr><td>Invasive Ductal (%)</td><td>78,53</td><td>82,66</td><td>74,49</td><td>79,55</td><td>85,00</td></tr>
<tr><td>Invasive Lobular (%)</td><td>13,09</td><td>5,78</td><td>15,30</td><td>6,65</td><td>15,00</td></tr>
<tr><td>Luminal A (%)</td><td colspan="2" rowspan="2">NA</td><td colspan="2" rowspan="2">NA</td><td>79,98</td></tr>
<tr><td>Luminal B (%)</td><td>20,02</td></tr>
</table>

Table S3: Descriptive sensitivity analysis of cohort grouping criteria (Δ>5% vs Δ>10%) and characterization of near-balanced cohorts. Abbreviations: LR: Low-Risk; HR: High-Risk.

Model	Variable	Beta	OR (95% CI)	p_value
Ki-67 continuous (per 1% increase)	Age (per year)	-0.04	0.96 (0.92–1.01)	0.0915
Ki-67 continuous (per 1% increase)	Pregnancies (per unit)	0	1.00 (0.62–1.61)	0.999
Ki-67 continuous (per 1% increase)	Tumor size: pT1c vs pT1ab	1.955	7.07 (1.38–47.66)	0.0269
Ki-67 continuous (per 1% increase)	Tumor size: pT2 vs pT1ab	3.778	43.72 (6.83–378.82)	0.000182
Ki-67 continuous (per 1% increase)	Nodal status: positive vs negative	1.648	5.20 (1.57–20.18)	0.0105
Ki-67 continuous (per 1% increase)	Ki-67 (1% increase)	0.111	1.12 (1.06–1.19)	0.000346
Ki-67 dichotomized (≥20% vs <20%)	Age (per year)	-0.041	0.96 (0.92–1.00)	0.057
Ki-67 dichotomized (≥20% vs <20%)	Pregnancies (per unit)	-0.086	0.92 (0.59–1.42)	0.7
Ki-67 dichotomized (≥20% vs <20%)	Tumor size: pT1c vs pT1ab	1.168	3.22 (0.75–17.09)	0.131
Ki-67 dichotomized (≥20% vs <20%)	Tumor size: pT2 vs pT1ab	3.305	27.24 (4.86–198.36)	0.000382
Ki-67 dichotomized (≥20% vs <20%)	Nodal status: positive vs negative	1.847	6.34 (1.85–27.37)	0.00651
Ki-67 dichotomized (≥20% vs <20%)	Ki-67 ≥20% vs <20%	1.983	7.26 (2.12–30.03)	0.00294

Table S4: Full multivariable logistic regression results.
Complete results of the multivariable logistic regression models including regression coefficients (β), odds ratios (OR), 95% confidence intervals (CI), and p-values.

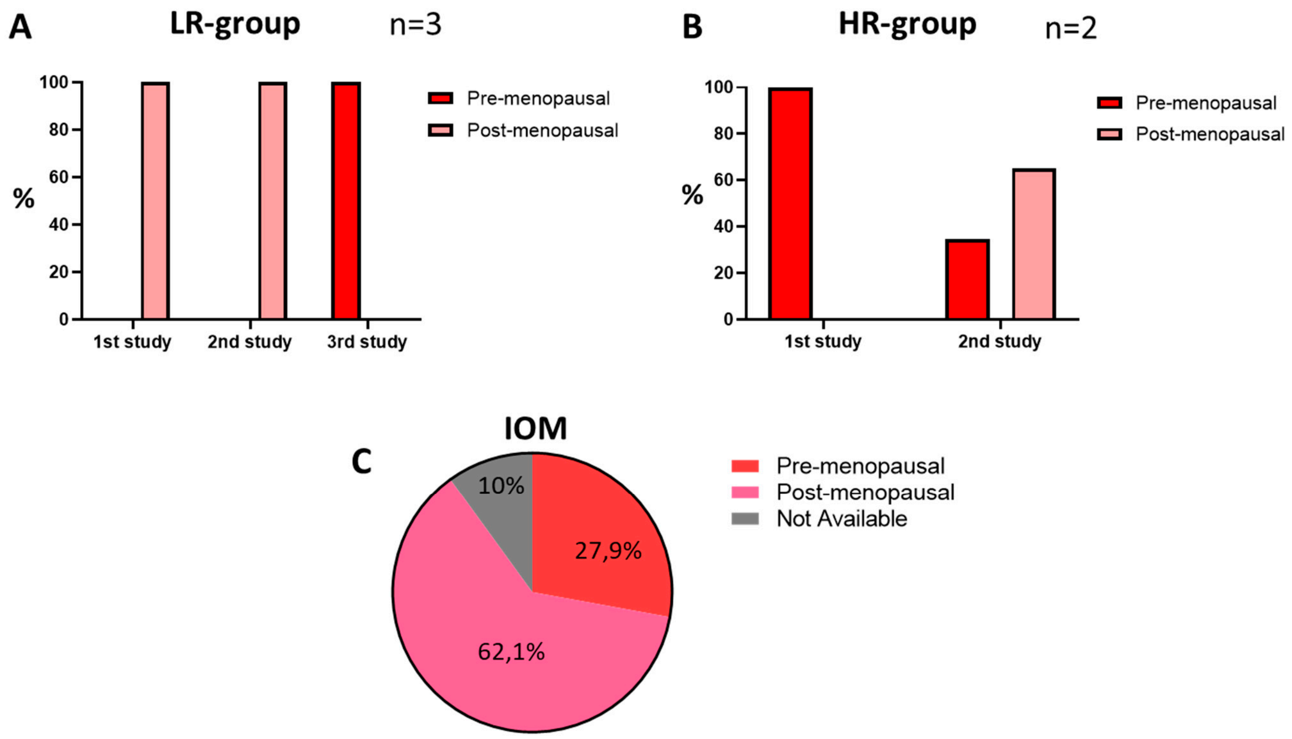

Figure S1: Menopausal status.

Bar charts of LR- (A) and HR-studies (B) providing menopausal status of their enrolled patients. Pie chart of IOM cohort (C). Abbreviations: LR: Low-Risk; HR: High-Risk; IOM: Istituto Oncologico del Mediterraneo. "%" symbol on the y-axis refers to the percentages of patients for each selected study. Calculations are unweighted.

Menopausal status	Low-Risk (%)	High-Risk (%)
Pre-menopausal	14 (36%)	25 (64%)
Post-menopausal	28 (32%)	59 (68%)
Not Available	6 (43%)	8 (57%)

Table S5: Table of numbers and percentages of LR/HR cases for the three indicated categories.

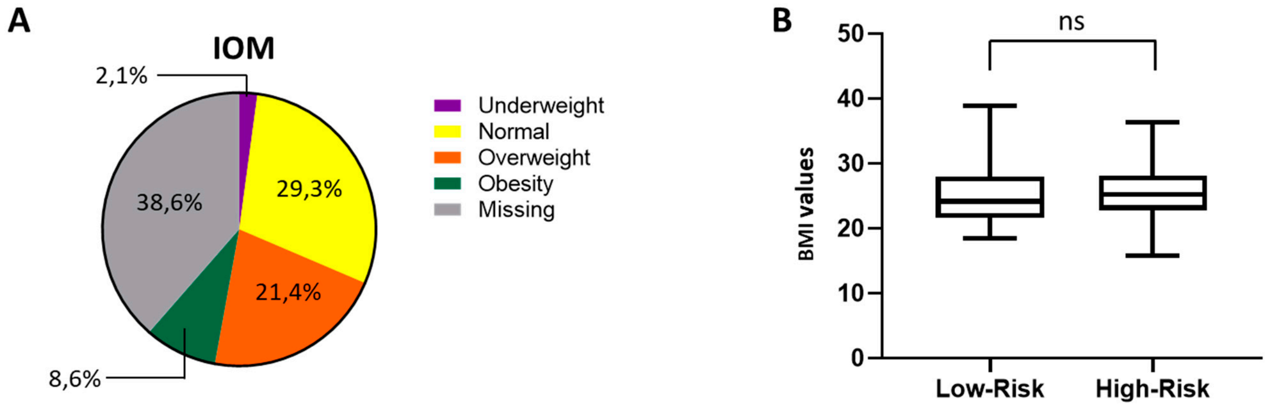

Figure S2: Body Mass Index.
Pie chart of IOM cohort stratification related to BMI values (A). Graphical representation of the statistical analysis of BMI LR/HR cases (B). Abbreviations: IOM: Istituto Oncologico del Mediterraneo; BMI: Body Mass Index; LR: Low-Risk; HR: High-Risk. “ns” symbol represents $p > 0.05$.

BMI status	Low-Risk (%)	High-Risk (%)
Underweight	1 (33%)	2 (67%)
Normal	14 (34%)	27 (66%)
Overweight	8 (27%)	22 (73%)
Obese	4 (33%)	8 (67%)
Not Available	21 (39%)	33 (61%)

Table S6: Table reporting the numbers and relative percentages of LR/HR-subjects in all defined categories. Abbreviations: LR: Low-Risk; HR: High-Risk.

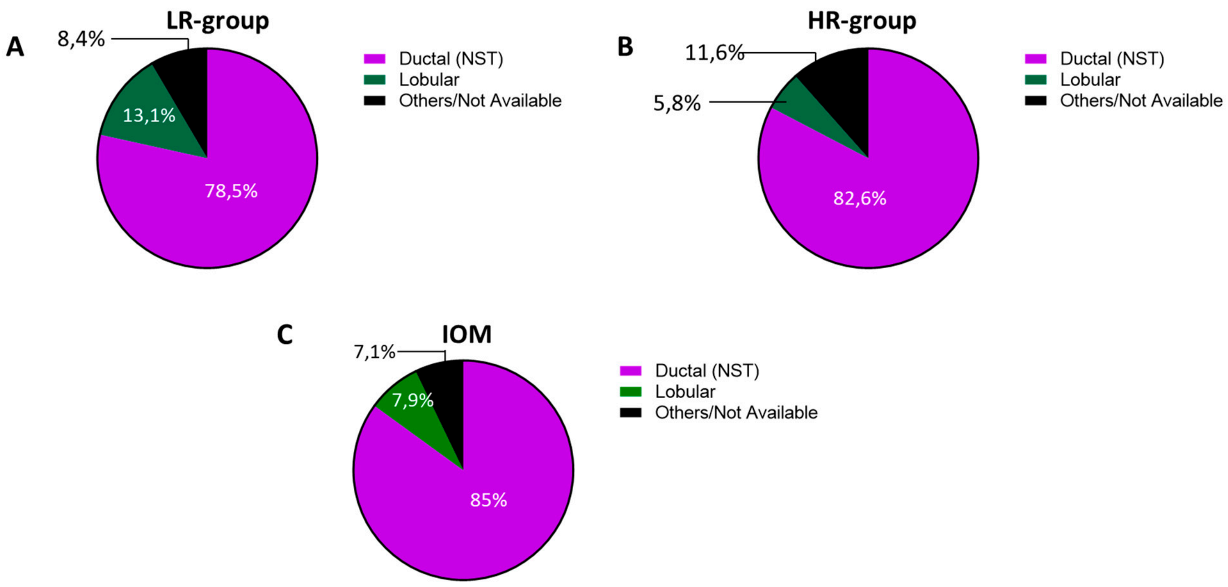

Figure S3: Histological subtypes.
Pie charts of histological subtypes percentages in LR- (A) and HR-groups (B). Pie chart of IOM cohort patients' stratification based on histological subtypes (C). Abbreviations: NST: non-special type; IOM: Istituto Oncologico del Mediterraneo.

	Invasive Ductal (NST) (%)	Invasive Lobular (%)	Others/Not Available (%)
Low-Risk (n=5)	78,53 (70,63 - 94,68)	13,09 (4,26 - 18,77)	8,48 (1,06 - 13,50)
High-Risk (n=3)	82,66 (77,61 - 88,89)	5,78 (0 - 13,30)	11,55 (5,20 - 22,39)

Table S7: Table reporting LR- and HR-groups percentages and ranges for histological subtypes. Abbreviations: NST: non-special type; LR: Low-Risk; HR: High-Risk.

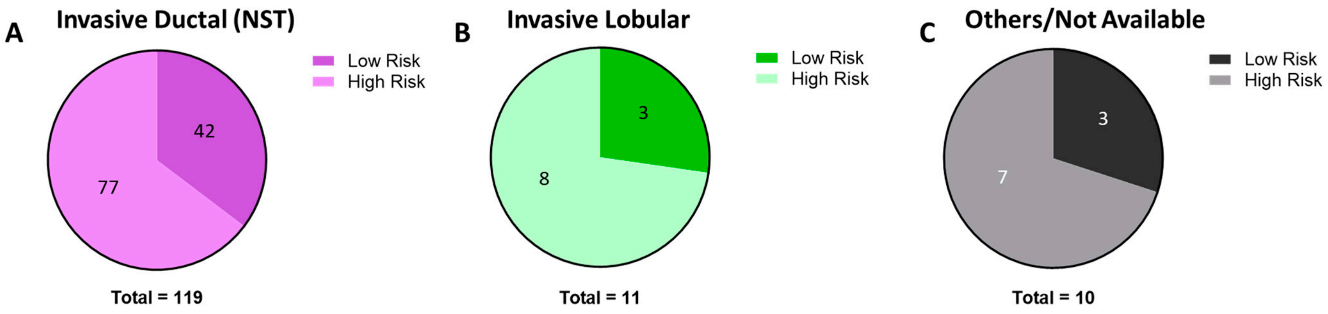

Figure S4: Histological subtypes of IOM cohort.
Pie charts of IOM cohort LR/HR-cases based on ductal (A), lobular (B) and others subtypes/not available data (C) discrimination. Abbreviations: NST: Non-special type; IOM: Istituto Oncologico del Mediterraneo; LR: Low-Risk; HR: High-Risk.

	Invasive Ductal (NST) (%)	Invasive Lobular (%)	Others/Not Available (%)
Low-Risk	42 (35%)	3 (27%)	3 (30%)
High-Risk	77 (65%)	8 (73%)	7 (70%)

Table S8: Table reporting the relative percentages of histological subtypes in all defined categories. Abbreviation: NST: non-special type.
